# Mucosal Immunity and B Cells in Teleosts: Effect of Vaccination and Stress

**DOI:** 10.3389/fimmu.2015.00354

**Published:** 2015-07-15

**Authors:** David Parra, Felipe E. Reyes-Lopez, Lluis Tort

**Affiliations:** ^1^Animal Physiology Unit, Department of Cell Biology, Physiology and Immunology, School of Biosciences, Universitat Autonoma de Barcelona, Cerdanyola del Valles, Spain

**Keywords:** MALT, stress, B-lymphocytes, mucosal vaccination, CMES

## Abstract

Fish are subjected to several insults from the environment, which may endanger animal survival. Mucosal surfaces are the first line of defense against these threats, acting as a physical barrier to protect the animal but also functioning as an active immune tissue. Thus, four mucosal-associated lymphoid tissues (MALTs), which lead the immune responses in gut, skin, gills, and nose, have been described in fish. Humoral and cellular immunity, as well as their regulation and the factors that influence the response in these mucosal lymphoid tissues, are still not well known in most fish species. Mucosal B-lymphocytes and immunoglobulins (Igs) are key players in the immune response that takes place in those MALTs. The existence of IgT as a mucosal specialized Ig gives us the opportunity of measuring specific responses after infection or vaccination, a fact that was not possible until recently in most fish species. The vaccination process is influenced by several factors, being stress one of the main stimuli determining the success of the vaccine. Thus, one of the major goals in a vaccination process is to avoid possible situations of stress, which might interfere with fish immune performance. However, interaction between immune and neuroendocrine systems at mucosal tissues is still unknown. In this review, we will summarize the latest findings about B-lymphocytes and Igs in mucosal immunity and the effect of stress and vaccination on B-cell response at mucosal sites. It is important to point out that a limited number of studies have been published regarding stress in mucosa and very few about the influence of stress over mucosal B-lymphocytes.

## Introduction

All animals may be subjected to different kinds of stressors. Stress response presents a number of mechanisms that share common pathways for most animals. Hence, the organism responds to a challenge that could be hazardous for its integrity. A complex network takes part in this response involving three main regulatory systems, such as neural, endocrine, and immune. In lower vertebrates, like fish, the neuroendocrine response under stress circumstances has been well described in numerous species and it involves several key hormones as important players in the response. Thus, sympathetic–chromaffin axis is first activated after stress by the central nervous system, producing catecholamines, which are rapidly released into circulation. Other stress hormones, such as corticosteroid releasing hormone (CRH), adrenocorticotropic hormone (ACTH) and cortisol, are secreted by the hypothalamic-pituitary-interrenal axis (1). Cortisol is the central corticosteroid in teleosts (2), and circulating levels of this hormone in plasma is the most common indicator of the degree of stress experienced by fish (3). However, in the rest of fluids in fish, such as the mucosal secretions of skin, gut, gills, or bile, little is known about cortisol levels, the effect of stressors in the composition of these secretions or the local responses triggered by stress. A recent publication has used one of these alternative matrixes to solve a major problem that stress studies have to face the detection of chronic stress circumstances in fish farms. Thus, thanks to the analysis of cortisol levels in scales, authors were capable to determine not only whether the fish was under stress at that moment but also if that fish was previously exposed to a stress situation (4).

**Figure 1:**
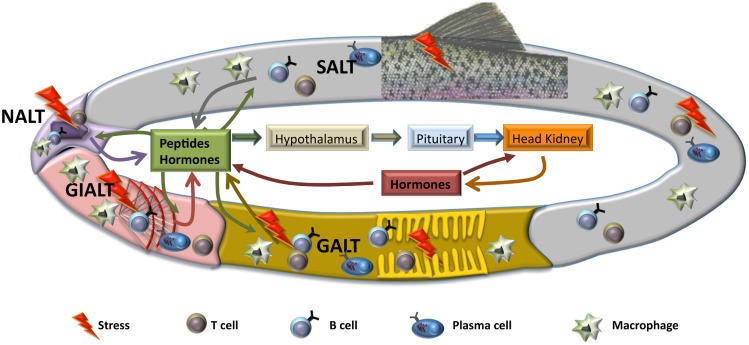
**Schematic representation of hypothetical local mucosal responses to stress**. After a stress situation, mucosal tissues produce several mediators, including hormones and other peptides, such as cytokines or chemokines, that will act on the rest of mucosal tissues, activating similar responses with the purpose of maintaining homeostasis. Also, triggered mucosal responses will lead to an activation of the hypothalamic–pituitary–interrenal (HPI) axis and the beginning of the neuroendocrine response at systemic level. Both local and systemic responses will be sustained through to a reciprocal feedback, until the insult has finished. SALT, skin-associated lymph tissue; GALT, gut-associated lymph tissue; NALT, nose-associated lymph tissue; GIALT, gill-associated lymph tissue.

The response of fish to stressors involves different mechanisms at different functional levels, from gene, molecular, and cellular to systemic and performance responses. The classical view considered that the stress response is the result of a previous perception of stressors by the sensors of the nervous system followed by activation of a neuroendocrine reaction in brain and pituitary, thus involving the secretion of catecholamines and corticosteroids that would generate the response in peripheral tissues to face the alarm situation and, at last, return to the basal condition. Assuming that these mechanisms are produced, part of the response is missing in this classical view, since some of the local responses and their influence in the overall activation of the stress reaction is not considered. Thus, the stressing agents may activate the local receptors and generate a first response in a particular tissue, mostly in the portals of entry, i.e., the surfaces that are in contact with the external environment, namely, gills, nose, gastrointestinal tract, and skin. The changes at these mucosal tissues will produce local alterations in specific tissue receptors and they may also produce messenger substances (hormones, cytokines, peptides) that will activate the overall physiological response. In this case, the consequence is that the perception is not produced at central and nervous level, but at the local and tissue level. For instance, pathogens, pollutants at low concentration or specific dietary components may trigger a local response that will induce a subsequent global neuroendocrine response when the alarm messengers will reach the brain, pituitary, or head kidney (Figure 1). In addition, when looking at the transcriptomic response to stressors in the portals of entry, such as gills or gut, results show an inespecific response involving different regulatory pathways, i.e., oxidative, immune, or endocrine (5).

The relationship between endocrine and immune system after stress has been widely described [reviewed in Ref. (6)]. Several studies have proved that under stress, the animal suffers an immune suppression that could eventually lead to an increase in susceptibility to pathogens and a reduction in vaccine protection (6–9). However, in fish, the mechanisms behind this immune suppression are not well known. Some effects of stress, mostly due to cortisol release, on immune responses described in fish are: increase of neutrophils, reduction of lymphocyte number and antibody responses (10–13), reduction of complement activity (14), and decrease in the production of some cytokines, such as TNF-alpha, TGF-beta, or IL-6 (15). The lack of tools to characterize and separate immune cell subsets has complicated enormously the analysis of the stress effects on specific cell populations. Thus, few studies have been published about the effect of stress over any of the immune cells, such as B or T lymphocytes. Moreover, to date, little is known about the effects of vaccines that target mucosal surfaces over B-lymphocytes. In this review, we will focus on the role of B-lymphocytes and the effect of both, stress and vaccines, on B-cell response at the mucosa surface.

## B-Lymphocytes and Mucosal Immunity in Teleost

B-lymphocytes are present in all vertebrates and their function as antibody-secreting cells (ASC) in adaptive immunity is conserved throughout evolution (16) (Table 1). Interestingly, B cells have innate features, such as phagocytic capability, natural antibodies secretion, or cytokine production, that are also conserved (17). Although agnathan vertebrates, such as lampreys, have a homologous cell type called VLR-B cells (18), gnathostome fish are the first animals in evolution to express immunoglobulins (Igs) and to posses B cells (16). The main function of B cells is the production of Ig, also called antibodies. Igs are constituted by heavy and light chains, and present a constant region, common to all the Igs of the same isotype and is made up by heavy chains, and one variable region which gives them the specificity, and it is made up by heavy and light chains. Teleosts express three different Ig heavy chains, capable of generating three Ig isotypes expressed on the B-cell surface: IgM, IgT (also called IgZ in some species), and IgD. Although different isotypes are present, teleosts lack class switch recombination (CSR) (19). Interestingly, the main enzyme implicated in this process, activation-induced cytidine deaminase (AID), is expressed in teleosts and even is capable of leading CSR process when transferred into a mouse (20). Thus, the different isotypes are produced due to the structure of rainbow trout (*Oncorhynchus mykiss*) and zebrafish (*Danio rerio*) heavy chain locus, which implies that the expression of IgM isotype blocks the generation of IgT/IgZ transcripts, and vice versa (21, 22). This has been confirmed in rainbow trout (23, 24) and zebrafish (25), which have a lineage of B cells uniquely expressing IgT or IgZ, respectively. In carp (*Cyprinus carpio* L.), two IgZ have been identified recently, IgZ1 and IgZ2, which have a different expression in systemic or mucosal compartments (26). This situation is very similar to that in humans, where two IgA are present and their expression vary depending on the tissue (27). Teleosts present two populations of IgD^+^ B cells: (1) IgD^+^/IgM^−^ B cells, observed only in channel catfish (*Ictalurus punctatus*) (28) and European rainbow trout (29); (2) IgD^+^/IgM^+^ B cells, which have been described in all teleost species analyzed thus far. Interestingly, in American rainbow trout IgD^+^/IgM^−^ population has not been described so far (30).

**Table 1:** **Mucosal immunity and B cells in teleost and mammals**.

	Teleost	Mammals
**MALT**		
Nomenclature	NALT (Nose), SALT (Skin), GIALT (Gills), GALT (Gut)	NALT (Nose), BALT (Bronchus), GALT (Gut), VALT (Genital)
Associated structures	Lamina propria, ILT	Lamina propria, lymph nodes, germinal centers
Resident immune cells	B cells, T cells, plasma cells, macrophages, neutrophils, rodlet cells	B cells, T cells, plasma cells, dendritic cells, macrophages, neutrophils.
Antigen-uptaking cells	M-cells?, dendritic cells?	M-cells, dendritic cells
**B LYMPHOCYTES**		
B cell subsets	IgM^+^IgD^+^; IgM^−^IgD^−^IgT^+^; IgM^−^IgD^+^	IgM^+^IgD^+^; IgM^−^IgD^−^IgA^+^; IgM^−^IgD^−^IgG^+^; IgM^−^IgD^+^
Secretory Igs	IgM, IgT/Z (main mucosal Ig)	IgM, IgD, IgG, IgA (main mucosal Ig)
Ig structure	IgM tetrameric	IgM pentameric
	IgT polymeric	IgA dimeric/monomeric
Transport Igs to lumen	pIgR	pIgR, FcRn
J-chain in Igs	No	Yes
CSR	No	Yes
Affinity maturation	Low	High
Phagocytic capacity	Yes (ND in mucosa)	Yes (ND in mucosa)

*Comparison of main features of mucosal-associated tissues and B cells in teleost and mammals*.

*MALT, mucosal-associated tissues; ILT, inter-brachial lymph tissue; pIgR, polymeric immunoglobulin receptor; FcRn, neonatal Fc receptor; ND, not determined*.

Out of these three immunoglobulin isotypes, secreted IgM and IgT are present in rainbow trout mucosal surfaces. IgM is the most common immunoglobulin in serum and mucus and the key player in systemic immune responses, whereas IgT is the main responder in mucosal surfaces (31). However, in some fish species, such as the channel catfish, IgT is not present, and IgM is the main responder in both compartments which seem to be integrated (32–34). Igs play a major role in adaptive immunity by recognizing the pathogen and helping with its destruction through various processes, such as complement activation and phagocytosis. Teleost Igs are secreted mainly by plasmablasts and plasma cells, which are located mainly within the head kidney (35). The main differences between these ASC are that plasmablasts can proliferate and they present low antibody secretion, while plasma cells are in a non-replicative state, they are terminally differentiated and posses a high capacity of antibody secretion (35). Characterization of these ASC populations, as well as early stages of B-cell development, has been performed using specific markers for B cells, such as HC mu, Pax5, or RAG1 (36, 37). Also, these ASC populations have been separated in several trout tissues thanks to their different size and density (38) and to their distinct expression of IgM on their surface (36). Teleost plasma cells can be divided into short-lived plasma cells (SLPC) and long-lived plasma cells (LLPC), which differ in their life span but also in their distribution, as LLPC are located only in the head kidney (39). Thus, it seems clear that teleost have all the components for mounting an efficient adaptive response mediated by B cells. However, the immunological memory in teleost is a matter of dispute. The fact that antibody response in fish shows poor anamnestic properties, meaning that antibody affinity increases marginally, compared to the logarithmic increases observed in mammals (40); also that very few studies about clonal expansion in teleost have been published; and finally that fish adaptive response is slower than in mammals and specific titers are not normally detected in fish until the third or fourth week after immunization (35), lead some authors to consider the idea of innate memory more feasible than adaptive memory in secondary response in teleost (41, 42). Nevertheless, affinity maturation in teleost B cells exists (43), as well as clonal expansion (44), and teleost are protected thanks to memory responses for several years post immunization (34), thus suggesting that both memories coexist in teleost fish and both contribute to fish protection against pathogens.

Apart from this adaptive role, B-lymphocytes have several innate capacities, such as phagocytosis, production of natural antibodies, and cytokine secretion. Although mammalian and fish B-lymphocytes share these innate capabilities, their importance in organism defense may not be comparable in some cases. For instance, whereas in mammals phagocytic B cells are confined to the peritoneal cavity and liver and represent a 5–15% of all B cells in these tissues (19, 45), in teleost fish, phagocytic B cells are present in all systemic compartments, such as blood, spleen, and head-kidney, and represent 60% of all B cells (23, 46). Thus, the innate role of B cells in fish seems to be much more important than that in mammals. It is worthy to note that dendritic cells are one of the main cells in the innate response in mammals, linking innate and adaptive immunity (47). The role of dendritic cells is vital for mammalian immune responses and their interaction with T cells is necessary for the beginning of the adaptive immune response. However, in teleost the existence of dendritic cells has been scarcely described and their function and location are still not well understood (48, 49). Thus, in contrast to B cells, fish dendritic cells are not as numerous as their mammalian counterparts and, so far, have not been described in all tissues. Hence, it is very tempting to hypothesize that fish B cells are carrying out part of the roles that dendritic cells perform in mammals. Indeed, the function of B-lymphocytes as a bridge between innate and adaptive responses might be predominant in teleost fish (50). As a result, the role of B cells in vaccination would be connected not only to the adaptive response as specific antibody-producing cells, but also would be key for the initial innate response. Consequently, the innate functions of B cells, which so far have been scarcely studied after a vaccination/infection process, will help to a great extent to the success of the vaccine.

Response to vaccines or challenges in fish, as well as in the rest of vertebrates, involves central and local responses. Thus, the head kidney, thymus, and spleen are the central immune organs responsible for leukocyte production, proliferation of T cells, and antigen capture in systemic compartment, respectively. Local response takes place in all tissues where immune defense based in innate immune factors, such as lysozyme, lectins, proteases or complement proteins, and cellular and humoral responses (antibodies) helps maintain the tissues free from pathogens. Therefore, both responses, central and local, are activated when facing an outbreak, but local responses can be working in a particular surface/tissue without yet an activation of the central organs. Mucosal responses in fish are an example of these independent local responses. Thus, mucosal surfaces are continuously acquiring information from the environment, processing it, and adapting themselves to maintain homeostasis necessary for animal survival. Any disruption in this homeostasis, i.e., a stress situation, will lead to an instability that could endanger animal health. The functions of these mucosal surfaces are various, from nutrient uptake to gas exchange or as immunological barriers (31, 51, 52). As immunological sites, mucosae are capable of mounting a robust immune response after a pathogen challenge in all vertebrates and some invertebrates too (31, 53–56). Despite the enormous morphological differences between species in vertebrates, all of them posses mucosal-associated lymphoid tissues (MALT) that control the immune response at mucosal site (Table 1). In teleost, four MALTs have been described: nose-associated lymphoid tissue (NALT), skin-associated lymphoid tissue (SALT), gill-associated lymphoid tissue (GIALT), and gut-associated lymphoid tissue (GALT). Common features of these four tissues would be: (1) the lack of organized lymphoid structures, such as lymphoid nodes or germinal centers, that lead to a disperse location of leukocytes; (2) the presence of secretory Igs in the mucus, which are transported into the lumen through a polymeric Ig receptor (pIgR); (3) the presence of a specialized mucosal immunoglobulin class, IgT/Z; and 4) the presence of commensal bacteria, some of them coated by Igs.

### Gut-associated lymphoid tissue

Mucus layer, which acts as a physical and chemical barrier, protects gut epithelium and therefore behaves as an important mechanism of innate defense that maintains tissue homeostasis (55, 57). Mucus is permeable to some macromolecules, i.e., the ones necessary for digestion, but also acts as an effective barrier to microorganisms (58). Its composition varies slightly amongst the different teleost species but is formed mostly by water (95%) and some glycoproteins, mainly mucins, which give the characteristic viscosity to the mucus. Other molecules are present in small quantities, including innate and adaptive immune factors, such as antimicrobial molecules or secretory Igs (9, 31, 55). Changes in mucins composition alter mucus structure and can compromise epithelium stability. Although their great importance, very few intestinal mucins have been described in teleost and when done, only at expression level (59).

Teleost gut contains two main populations of immune cells: (1) lamina propria leukocytes (LPLs), which include a variety of resident immune cells, such as granulocytes, macrophages, lymphocytes, and plasma cells; (2) intraepithelial lymphocytes (IELs), composed by T cells and some B cells located among epithelial cells. These immune cells together with epithelial cells, goblet cells, and neuroendocrine cells produce and regulate gut immune responses.

In the intestine of rainbow trout, two populations of B cells, differing in the expression of IgM or IgT on their surface, have been described (23, 60). They are both located in the lamina propria and can infiltrate the epithelium after infection (23) or vaccination (61). As resident cells, IgT^+^ is the dominant population of B cells (54% of the total gut B cells) and are the main responders against a gut parasite, *Ceratomyxa shasta*, in which response mucosal IgM is not implicated (23). Thus, specific IgT antibodies against this parasite have been obtained in gut mucus, while IgM titers were restricted to serum (62). Thus, IgT, same than IgA in mammals, is the main responder in gut and, as mentioned above, the first specialized mucosal immunoglobulin in vertebrates. However, immunity against viral infection and immune response after vaccination against virus in intestine seem to implicate IgM as well (61). Although IgT is the main B-cell subset in intestine of several teleost, the presence of resident IgT-plasma cells in teleost gut has not been demonstrated so far. On the contrary, IgM-plasma cells have been identified in the intestine of several teleost, including rainbow trout (63) and carp (62, 64). In fish lacking IgT, such as catfish, resident B cell has been described in all the segment of intestine (65). No IgD^+^/IgM^−^ B cells or secreted IgD has been observed in intestine or gut secretions so far.

### Skin-associated lymphoid tissue

The skin is the largest mucosal tissue in teleost, and its basic structure is similar in all species, although some differences exist, like the catfish skin, which presents no scales. Similar to that in mammals, teleost skin is constituted by an epidermis and a dermis, although two important differences exist compared to mammalian skin: (1) the outermost layer of the skin is not keratinized in fish; (2) the presence of mucus-secreting cells in the epidermis of fish. These two features characterize teleost skin as a mucosal tissue. Some of the molecules that confer antimicrobial properties to the skin mucus are lysozyme, complement components, lectins, proteolytic enzymes, and Igs (66). So far, secreted IgM and IgT have been described in mucosal skin secretions in fish (24, 32), while presence of IgD has not been reported. IgM is the most abundant immunoglobulin in skin mucus, although, when IgT is present, IgT/IgM ratio is much higher in skin mucus than in serum. Microbiota in the skin is coated by Igs, with IgT showing higher ability to coat bacteria compared to IgM (24). Interestingly, Ig concentration in skin is different depending on the area of the body. For example, Ig levels in channel catfish are higher in lateral skin than in the fins (67). As described in gut, when present, IgT^+^ B cells represent the main B-cell subset in the skin and, together with IgM^+^ B cells, are located in the epidermis in trout (24). IgM^+^ B cells were previously observed in the epidermis of carp and spotted wolfish (*Anarhichas minor*) (68). Existence of plasma cells has been reported in channel catfish skin (33) and recently suggested in trout (24).

In skin, B-cells response in trout against the parasite *Ichthyophthirius multifiliis* (Ich) is very similar to that described before in gut. Thus, an important increment in IgT^+^ B cells and that in secretory IgT levels were detected in skin mucus. Also, specific antibodies against parasite, mainly IgT, were observed in mucosal secretion of skin (24). Interestingly, in this article, some IgT titers were observed in serum, indicating a possible production of IgT outside the skin or a role of specific IgT in non-mucosal tissues (24). In channel catfish, IgM titers have been measured in skin after immunization against the same protozoan parasite (32, 69). More importantly, Xu et al. proved the presence of ASC for IgM in catfish skin by using skin explants experiments (69).

### Gill-associated lymphoid tissue

Gills are in charge of oxygen exchange with the environment. Their structure is very similar to the one described for alveolar sacs in mammals. However, the number of pathogens and antigens in the water is higher than that found in the air, and gills are exposed continuously to them. Interestingly, an accumulation of immune cells has been described in trout gills in the interbrachial lymphoid tissue (ILT) (70, 71). This exceptional aggregation, resembling to a lymph node, is only present in gills, while in the rest of trout MALTs, no aggregations have been observed so far. This lymphoid tissue is constituted mainly by T cells and some scattered B cells (71). Lymph nodes in mammals are essential for mounting a rapid and precise immune response, thus, the fact that a similar structure have been observed in gills indicate the importance of this tissue in fish protection and the necessity of a sophisticated immune response to avoid pathogen entry. Each side of the animal has four arches that develop into a primary lamellae and secondary lamellae. Presence of B cells in gills has been described in naïve fish and after infection. As described in mammalian respiratory tract (72), gills in trout and catfish present an IgM^−^IgD^+^ B-cell population (28–30). The roles of this population and secreted IgD are still unknown in teleost or mammals.

As specialized mucosal immunoglobulin implicated in gut and skin immune response, IgT acts as main responder in gills too (73). IgT^+^ B cells are the main population of B cells in gills and IgT constitutes the main humoral component after infection. *I. multifiliis* (Ich) infection produces an increase in IgT^+^ B-cell number, as well as an increment in total protein level. Specific IgT against Ich was also observed in gill mucus but not in serum (73). Interestingly, IgT and IgM binding to Ich trophons have been observed in the gills as early as 2 h post-infection, indicating the possible role of Igs in the innate response against parasites (74). Also specific-IgM titers have been observed in trout gills after bacterial infection (75).

### Nose-associated lymphoid tissue

Nasopharynx-associated tissue is considered the first line of defense to airborne pathogens in mammals, and it has also been described in trout (76). Structure of NALT is similar to that in mammals, apart from the fact that there are no organized structures. As in the other teleost MALTs, IgT seems to represent the main immunoglobulin in nasal mucosa, as it covers a large number of bacteria (76). Interestingly, after nasal vaccination, IgM is the main responder, although it has been measured only at expression level (77).

## Mucosal Vaccination and B-Cell Response

As first line of defense, MALTs are an important target for vaccine development and formulation. Although formulation of fish vaccines has improved enormously in the last decade, it is imperative to design novel vaccination methods able to stimulate the immune response in systemic and mucosal compartments and to protect fish from threats to keep animal welfare. Analyses of vaccine success have been focused on gene expression analysis, post-challenge accumulative mortality, and, in some cases, pathogen load measurement. Levels of specific antibodies against pathogens in systemic compartments have also been widely used as indicators of successful immune response after vaccination. However, so far, few studies have focused on the evaluation of vaccines and their effect on the immune response in mucosa, including B-cell populations and specific antibody responses. In fish, the immunization routes used are intraperitoneal and intramuscular (IM) as systemic routes, and immersion (mainly bath), oral, and nasal, as mucosal routes.

Oral vaccines present some downsides, such as that they may induce tolerance (78) and that they need to be protected to escape digestion. However, and despite these limitations, fish oral vaccination offers important advantages when compared to other vaccination routes. Thus, oral vaccination is simple, effortless, cost-effective, and it is a stress-free immunization method. Based on this, several efforts have been made to test several oral vaccines to exploit the potential of mucosa to mount an effective immune response against a pathogen without the side effect of provoking stress in fish.

Studies in oral vaccines have been focused on the evaluation of the fish immune response at serological level. Thus, in rainbow trout, oral vaccination with *Lactobacillus casei* (79) and a VP2 DNA vaccine (80) against IPNV, showed an increment in serum IgM titer with neutralizing activity and viral load reduction. Apart from serum, which is a fundamental indicator of the systemic response, mucosal responses have been measured in some studies. Thus, an increase in IgM^+^ and IgT^+^ IELs at pyloric caeca has been observed after the use of an alginate encapsulated DNA vaccine against IPNV, thus supporting the relevance of B cells in the mucosal response after vaccination (61). However, whether this cellular increment is produced by an infiltration of B cells from blood or by an *in situ* cell proliferation is still unknown. Regarding this B-cell migration mechanism, few but promising advances have been done about the recruitment of B cells in mucosa. In sea bass (*Dicentrarchus labrax*, L.), it has been described that the CCL25/CCR9 ligand/receptor system appears to be a crucial step in IEL homing to the hindgut epithelium to provide protection at mucosal level (81), while in rainbow trout it was demonstrated that several chemokines, such as CK9 or CK10, have chemotactic capacities for B-cell recruitment to the intestine after immunization (80).

Non-conventional oral vaccine strategies have been developed but the consequences on the mucosa surface still need to be elucidated. For example, one vaccination model using a viral G protein linked to a gut adhesion molecule (LTB) in potato tubers was evaluated in carp, evoking a systemic immune response (82). Similar results were observed in Rock bream (*Oplegnathus fasciatus*) when administering an iridovirus antigen expressed in transgenic rice callus (83).

Taken together, it seems that the oral vaccine promotes the activation of the immune system, including B cells recruitment and humoral response mediated by IgM and IgT.

Recently, the nasal route has been reported as a new vaccine administration method to promote the mucosal immunity, showing a clear immune response by histological analysis (77). Despite these advances, more knowledge should be generated to understand the mechanisms underlying the immune response in fish NALT. Although nasal immunization is capable of protecting fish against pathogens, its application is doubtful in aquaculture industry due to its difficulty of administration. However, during immersion/bath vaccination, antigens enter through all four mucosal sites and, therefore, NALT is also implicated in the vaccination success. Thus, immersion vaccination would be the best administration route to induce mucosal responses, as it would likely produce a broader mucosal response. Hence, regarding gills response, immersion vaccination using killed bacteria in yellow croaker (*Pseudosciaena crocea*) and sea bass produces a drastic increment in ASC in gills (84, 85). Interestingly, in yellow croaker, the presence of specific IgM ASCs 7 days after vaccination was detected only after immersion vaccination, but not after oral or intraperitoneal injection (85). In concordance with these data, another report in European eel showed specific IgM titers in gills shortly after vaccination by immersion against *Vibrio vulnificus*, with a peak at day 3 after vaccine administration (86). As aforementioned, B cells present innate features that can influence immune response after vaccination. Thus, whether this IgM was specific or a result of natural antibodies secretion with polyclonal qualities was not clarified.

IgM responses in skin after immersion vaccination have been scarcely described. In many cases immunization was not able to induce such responses and, when present, specificity was low (31). Early studies with single antigens, such as dinitrophenylated-horse serum albumin (DNP), demonstrated the presence of specific antibodies in serum and skin mucus after bath vaccination (87). Similar data were obtained in European eel after vaccination with *V. vulnificus* by different routes, including bath (88). Specific skin IgM was described regardless the delivery route.

It seems obvious that the route of vaccination will determine the location of the primary immune response, and that a disparity in the response will be observed when the same antigen is administered by more than one route. Thus, differences between oral and IM injection were observed in carp when a supernatant fraction of a *Vibrio anguillarum* bacterin was encapsulated in alginate microparticles and administered as oral vaccine in food, observing that mucosal IgM^+^ plasma cells appeared to be present in gut and gills after oral vaccination and absent after IM injection in carp (64). It has recently been shown in rainbow trout that IgM level in serum, gill, and skin mucus increased significantly by 28 days after ip immunization with an attenuated *Flavobacterium psychrophilum* strain in rainbow trout, while no significant increase in IgM level was observed when fish were immunized by anal intubation or immersion (75). In the same study, expression levels of secretory IgD and IgT were significantly upregulated in gills of fish immunized by immersion, and in intestine of fish immunized by anal intubation route (75), reinforcing the role of IgT specialized in mucosal immunity (23, 73) and proposing IgD as a candidate to protect fish at mucosal surfaces. However, as aforementioned, the role of IgD in the mucosal response is still unknown. Another example is the vaccination of grouper larvae (*Epinephelus coioides*) with inactivated betanodavirus administered by bath and oral route, which showed an increased IgM gene expression in all analyzed tissues regardless of the administration route whereas IgT expression was dependent on the route (89). Thus, IgT increased in skin and gills but remained unchanged in gut after bath immunization, while an increment in its expression was detected in skin and gut but not in gills after oral administration.

Although antibody responses are recognized as excellent markers for vaccine success, an increase in IgM after immunization is not always associated with immune protection. Thus, a live attenuated *V. anguillarum* vaccine candidate was administered by immersion to zebrafish and observed that IgM serum levels against virulent *V. anguillarum* in the vaccinated group did not rise significantly following infection while in the non-vaccinated group the IgM response was increased. Interestingly, the gene expression in spleen of pro-inflammatory cytokine IL-1β and IL-8, together with the anti-inflammatory cytokine IL-10 were upregulated in non-vaccinated fish when compared to vaccinated fish, suggesting that the vaccine controlled the inflammatory response triggered by the infection (90). Therefore, immune-associated gene expression analysis is relevant to understand the immune protection mechanism conferred by vaccines.

## Mucosal Immunity, B Cells, and Stress

A major problem in aquaculture is the overpopulation of the fish farms that may lead to an important increase in the stress level of the animals. Despite the aforementioned advances, which have been made in the knowledge of mucosal immunity in recent years (23, 24, 78), the effects of stress at mucosal level in fish have been poorly described. Thus, the consequences of different stressors on the immune responses have been shown mainly in the systemic compartment, i.e., blood, head kidney, spleen, or liver. These reports have shown that stress affects directly the fish immune system due to immunosuppression and thus exposing fish to an increased susceptibility to disease (6, 91).

In gut is where we can find most data regarding the influence of stress in the immune system. Gut epithelia homeostasis is vital for animal survival. Stress produced by high density rearing in Atlantic salmon (*Salmo salar*) leads to an alteration in intestinal mucosa permeability which compromises animal welfare (92). Also, stress produced by chronic hypoxia in salmon produces an alteration in intestinal homeostasis, altering GALT responses by reducing, among others, IL-1β and IFN-γ expression, together with an alteration in IL-10 production and in antiviral responses (93, 94). This reduction in antiviral response due to cortisol in intestine could lead to an increase in IPNV infection in Atlantic salmon (95). Also, in a study at proteomic level in the digestive tract in rainbow trout, it was observed that short-term starvation resulted in a decrease in the concentration of serine protease inhibitors, which protect intestinal epithelia from enzymatic damage, the concentration of leukocyte elastase inhibitor (LEI) and transferrin in the anterior intestinal epithelia that would increase susceptibility of epithelial cells to enzymatic damage from serine proteases (96). Therefore, the stress condition promotes an overall decrease in the concentration of inhibitors that protect epithelia from enzymatic damage and compromises the ability of the intestinal epithelium to avoid bacterial infection in the anterior intestine of rainbow trout. In this frame, one fundamental element responsible for preserving the integrity of the mucosal barrier are the tight junctions (TJ), which correspond to the apical most junctional complex in many types of epithelial and endothelial cells. Variations in these TJ were observed after acute stress in rainbow trout gastrointestinal tract, where an ultrastructural study showed a widening of the TJ between enterocytes in the midgut (97). Recently, it has been reported that stress due to transportation affects the TJ-associated proteins expression pattern, observing an up-regulation of occludin and claudin in rainbow trout skin and its association with an effective alternative to protect the epithelial barrier against the increased skin-associated bacterial number in post-transport stressed fish (98). The modulation of gene expression of TJ-associated proteins has been also reported in gills, where some claudin isoforms in puffer fish were upregulated in response to cortisol treatment *in vitro* (99). Therefore, these data suggest a correlation between stress, cortisol level, TJ gene expression, and its association with an effective alternative to protect the epithelial barrier. Thus, certain diseases initiated at the mucosal surfaces, might promote an increase of cortisol and the expression of tight-junction genes as means of response against pathogens.

Another set of actors in the epithelial cell integrity are mucins, O-glycosylated glycoproteins present on the apex of all wet-surfaced epithelia. Characterization of mucin gene expression at mucosal level has been reported in gilthead seabream (*Sparus aurata*). When infected with a myxozoan parasite, *Enteromyxum leei*, an overall decrease in mucin gene mRNA levels was evident at the posterior intestinal segment, suggesting that the mucins alteration may affect the intestine functional integrity (59).

Very importantly, fish exposed to stress are affected by changes in the intestinal microbiota, both in quantity and also diversity. Thus, salmonid microbiota is altered by handling stress, a fact that can affect immune responses against pathogens and compromise gut homeostasis (100). Elucidation of the effect of stressors on bacterial populations in mucosal surfaces is relevant, because stress may cause the elimination of existing microbiota resulting in an imbalance of functional populations, and also due to the reduction of protecting mucus.

Several studies have also focused on the fish skin mucosa as one of the first line of defense against pathogen, which, as mentioned above, contains innate immune factors such as lectins, proteases, and antibacterial agents on its mucus (101–103). Interestingly, Vatsos and collaborators showed an increase in the number of mucus-producing cells in stressed sea bass skin (104). This evidence further supports the idea that the fish mucosal barrier is an important sensor for monitoring stress. Similar results were obtained in rainbow trout fed with low doses of cortisol, which presented a surprisingly reduction in the number of parasites, *Argulus japonicus*, suggesting that the increment of mucus-producing cells could be one of the reasons for this reduction (105). In the light of these data, a very interesting hypothesis is that low levels of cortisol might be beneficial in rejecting parasites and possibly other pathogens. Similar to what was described in intestine, acute stress by hypoxia or overcrowding, produced changes in bacteria communities in brook charr (*Salvelinus fontinalis*) skin, increasing the amount of pathological bacteria among those communities (106). At immunological level, cortisol is capable of suppressing the expression of several genes related to antigen presentation, as well as downregulating B- and T-cell activation, inflammatory responses, and antiviral responses in salmon skin (5).

Few studies have been published regarding the effect of stressors on gills in fish. Several of these works have focused in the adaptation of fish to marine water, as it occurs with salmonids. Thus, cortisol acts in synergy with other hormones to increase seawater tolerance, through an upregulation of cortisol receptors in gills, an increase in Na^+^/K^+^-ATPase activity in this organ and a regulation of gill chloride cells (107–110). Also environmental stressors, such as temperature changes, have been illustrated that reduce IgM expression in gills (111). The effect of stressors or exogenous cortisol over NALT secretions or cell populations has not been studied so far.

It seems clear that new efforts must be made to understand the effect of stress upon fish mucosal immunity. These new insights will allow us to determine the factors that are involved in situations in which both factors, stress and immunity, are crucial to ensure the effectiveness of treatments to improve the health status in fish, such as vaccines.

Regarding B cells, several studies performed in fish have focused on the effect of stress in B-cell response at systemic compartment. Thus, it has been observed that stress reduces the number of circulating B-lymphocytes, and decreases the antibody response after immunization *in vivo* (112). Also, a reduction in IgM levels and an increased susceptibility to nodavirus was reported together with high plasma cortisol level in sea bass stressed by water temperature variation (13), indicating that the stress induces a suppression effect on the fish immune system likely due to the increased levels of cortisol. As the main hormone produced in stress situations in fish, studies *in vivo* and *in vitro* testing the effect of cortisol over the immune system have been widely used as model to understand the stress process in teleosts. In carp, cortisol administration induces B-cell apoptosis and reduces their proliferation (10, 113). According to this, a reduction in immunoglobulin secretion, IgM mRNA transcription and also ASC number after cortisol treatment have been described in rainbow trout (114), winter flounder (*Pseudopleuronectes americanus*) (115), fugu (*Takifugu rubripes*) (116), and carp (117). Interestingly, cortisol increment in carp after temperature-related stress does not reduce antibody production or ASC in the animal (118). These last data indicate that, although cortisol is the main hormone implicated in stress, immune regulation by neuroendocrine system is more complex than just cortisol release and signaling through its receptors, and other components participate to control immune system in such situations.

In mucosal surfaces, very few studies have been published describing the effect of stress over mucosal B cells and most of them at expression level. Thus, environmental stressors, such as temperature changes, have been illustrated to reduce IgM expression in gills in grouper (111). In another study, cortisol has been described that reduces the expression of genes related to B-cell activation in trout skin (5).

Importantly, as a mucosal immunoglobulin in mammals, sIgA is used as a marker for stress situations, and its levels varies in mucosal surfaces depending of the stress (119–121). Whether sIgT/Z, as mucosal specialized Igs in teleost, alter their secretion after stress and could be a marker for stress in fish is still unknown.

## Concluding Remarks

As we have discussed above, the knowledge on mucosal responses in teleosts at many levels, including endocrinological and immunological, is far from clarifying the mechanisms implicated in the maintenance of homeostasis at mucosal sites. Regarding the endocrine response, the existence of mucosal endocrine cells has been described in gut (9), gills (122), and skin (123), and cortisol has been defined as component of skin and gut mucus (124). Thus, endocrine contribution is, without a doubt, relevant for several of the processes that take part in the local response at mucosal tissues. As mentioned before, all mucosal tissues described in fish posses MALT that controls immune response. Although in the last years important advances have been made in respect to mucosal immunity such as the detection of salmonid ILT (70), the discovery of IgT as mucosal immunoglobulin (23) or the identification of a functional NALT (76), links between immune and endocrine systems in gut, skin, gills, or nose have still not been unraveled. Even in systemic compartment where it is widely described that stress influences immune response (6), our information is incomplete, as most of the data concerning stress focuses in gene expression regulation and a very limited number of studies regarding cellular response or changes at protein level after a stress situation have been published. Thus, it seems clear that interrenal cells are the main cortisol producers, but it is still not known whether these cells capable of generating cortisol are only present in the head kidney or also, although unlikely, in other peripheral tissues. This possibility of local synthesis of cortisol at mucosal sites would have a clear parallelism with the immune system, which is capable of mounting local responses after a stimulus. In this vein, the concept of common mucosal immune system (CMIS), which suggests that an initial response in one mucosal place will generate similar response in the other mucosal tissues, is believed to happen in fish but it is not yet demonstrated in all mucosae (31). Hence, it is tempting to hypothesize the existence of a common endocrine system (CMES), term that would describe the local stress responses in fish in a particular mucosal surface which would affect the other mucosal tissues independently on the activation of the central organs. However, as mentioned above, the production of cortisol in gut, skin, gills, or nose is highly improbable, therefore other messenger molecules could be produced in one mucosa and influence the response in other mucosae (Figure 1). Thus, more in depth studies are needed to determine how stress affects the mucosa and the cells that conform it, and to show the presence or absence of the CMES and its effect over the CMIS.

## Conflict of Interest Statement

The authors declare that the research was conducted in the absence of any commercial or financial relationships that could be construed as a potential conflict of interest.

## References

[1] Barton BA. Stress in fishes: a diversity of responses with particular reference to changes in circulating corticosteroids. Integr Comp Biol (2002) 42:517–25. 10.1093/icb/42.3.517 21708747

[2] Barton BA Iwama GK Physiological changes in fish from stress in aquaculture with emphasis on the response and effects of corticosteroids. Ann Rev Fish Dis (1991) 1:13–26.

[3] Wendelaar Bonga SE The stress response in fish. Physiol Rev (1997) 77:591–625. 9234959 10.1152/physrev.1997.77.3.591

[4] Aerts J Metz JR Ampe B Decostere A Flik G De Saeger S. Scales tell a story on the stress history of fish. PLoS One (2015) 10:e0123411. 10.1371/journal.pone.0123411 25922947 PMC4414496

[5] Krasnov A Skugor S Todorcevic M Glover KA Nilsen F. Gene expression in Atlantic salmon skin in response to infection with the parasitic copepod *Lepeophtheirus salmonis*, cortisol implant, and their combination. BMC Genomics (2012) 13:130. 10.1186/1471-2164-13-130 22480234 PMC3338085

[6] Tort L. Stress and immune modulation in fish. Dev Comp Immunol (2011) 35:1366–75. 10.1016/j.dci.2011.07.002 21782845

[7] Saeij JPJ Verburg-Van Kemenade LBM Van Muiswinkel WB Wiegertjes GF. Daily handling stress reduces resistance of carp to *Trypanoplasma borreli*: in vitro modulatory effects of cortisol on leukocyte function and apoptosis. Dev Comp Immunol (2003) 27:233–45. 10.1016/S0145-305X(02)00093-9 12590974

[8] Baschant U Tuckermann J. The role of the glucocorticoid receptor in inflammation and immunity. J Steroid Biochem Mol Biol (2010) 120:69–75. 10.1016/j.jsbmb.2010.03.058 20346397

[9] Beck B Peatman E Mucosal Health in Aquaculture. San Diego, CA: Academic Press (2015).

[10] Weyts FAA Flik G Rombout JHWM Verburg-Van Kemenade BML. Cortisol induces apoptosis in activated B cells, not in other lymphoid cells of the common carp, *Cyprinus carpio* L. Dev Comp Immunol (1998) 22:551–62. 10.1016/S0145-305X(98)00033-0 9877436

[11] Wojtaszek J Dziewulska-Szwajkowska D Lozinska-Gabska M Adamowicz A Dzugaj A. Hematological effects of high dose of cortisol on the carp (*Cyprinus carpio* L.): cortisol effect on the carp blood. Gen Comp Endocrinol (2002) 125:176–83. 10.1006/Gcen.2001.7725 11884063

[12] Engelsma MY Hougee S Nap D Hofenk M Rombout JHWM Van Muiswinkel WB Multiple acute temperature stress affects leucocyte populations and antibody responses in common carp, *Cyprinus carpio* L. Fish Shellfish Immunol (2003) 15:397–410. 10.1016/S1050-4648(03)00006-8 14550666

[13] Varsamos S Flik G Pepin JF Bonga SEW Breuil G. Husbandry stress during early life stages affects the stress response and health status of juvenile sea bass, *Dicentrarchus labrax*. Fish Shellfish Immunol (2006) 20:83–96. 10.1016/j.fsi.2005.04.005 15961320

[14] Sunyer JO Gomez E Navarro V Quesada J Tort L Physiological responses and depression of humoral components of the immune system in gilthead sea bream (*Sparus aurata*) following daily acute stress. Can J Fish Aquat Sci (1995) 52:2339–46. 10.1139/f95-826

[15] Castillo J Castellanal B Acerete L Planas JV Goetz FW Mackenzie S Stress-induced regulation of steroidogenic acute regulatory protein expression in head kidney of gilthead seabream (*Sparus aurata*). J Endocrinol (2008) 196:313–22. 10.1677/JOE-07-0440 18252954

[16] Parra D Takizawa F Sunyer JO Evolution of B cell immunity. Ann Rev Anim Biosci (2013) 1:65–97. 10.1146/annurev-animal-031412-103651 25340015 PMC4203447

[17] Sunyer JO. Fishing for mammalian paradigms in the teleost immune system. Nat Immunol (2013) 14:320–6. 10.1038/ni.2549 23507645 PMC4203445

[18] Boehm T Mccurley N Sutoh Y Schorpp M Kasahara M Cooper MD VLR-based adaptive immunity. Annu Rev Immunol (2012) 30:203–20. 10.1146/annurev-immunol-020711-075038 22224775 PMC3526378

[19] Parra D Rieger AM Li J Zhang YA Randall LM Hunter CA Pivotal advance: peritoneal cavity B-1 B cells have phagocytic and microbicidal capacities and present phagocytosed antigen to CD4+ T cells. J Leukoc Biol (2012) 91:525–36. 10.1189/jlb.0711372 22058420 PMC3317272

[20] Barreto VM Pan-Hammarstrom Q Zhao Y Hammarstrom L Misulovin Z Nussenzweig MC. AID from bony fish catalyzes class switch recombination. J Exp Med (2005) 202:733–8. 10.1084/jem.20051378 16157688 PMC2212934

[21] Danilova N Bussmann J Jekosch K Steiner LA. The immunoglobulin heavy-chain locus in zebrafish: identification and expression of a previously unknown isotype, immunoglobulin Z. Nat Immunol (2005) 6:295–302. 10.1038/ni1166 15685175

[22] Hansen JD Landis ED Phillips RB. Discovery of a unique Ig heavy-chain isotype (IgT) in rainbow trout: implications for a distinctive B cell developmental pathway in teleost fish. Proc Natl Acad Sci U S A (2005) 102:6919–24. 10.1073/pnas.0500027102 15863615 PMC1100771

[23] Zhang YA Salinas I Li J Parra D Bjork S Xu Z IgT, a primitive immunoglobulin class specialized in mucosal immunity. Nat Immunol (2010) 11:827–35. 10.1038/ni.1913 20676094 PMC3459821

[24] Xu Z Parra D Gomez D Salinas I Zhang YA Von Gersdorff Jorgensen L Teleost skin, an ancient mucosal surface that elicits gut-like immune responses. Proc Natl Acad Sci U S A (2013) 110:13097–102. 10.1073/pnas.1304319110 23884653 PMC3740891

[25] Schorpp M Bialecki M Diekhoff D Walderich B Odenthal J Maischein HM Conserved functions of Ikaros in vertebrate lymphocyte development: genetic evidence for distinct larval and adult phases of T cell development and two lineages of B cells in zebrafish. J Immunol (2006) 177:2463–76. 10.4049/jimmunol.177.4.2463 16888008

[26] Ryo S Wijdeven RH Tyagi A Hermsen T Kono T Karunasagar I Common carp have two subclasses of bonyfish specific antibody IgZ showing differential expression in response to infection. Dev Comp Immunol (2010) 34:1183–90. 10.1016/j.dci.2010.06.012 20600275

[27] Woof JM Russell MW. Structure and function relationships in IgA. Mucosal Immunol (2011) 4:590–7. 10.1038/mi.2011.39 21937984

[28] Edholm ES Bengten E Stafford JL Sahoo M Taylor EB Miller NW Identification of two IgD+ B cell populations in channel catfish, *Ictalurus punctatus*. J Immunol (2010) 185:4082–94. 10.4049/jimmunol.1000631 20817869

[29] Castro R Bromage E Abos B Pignatelli J Gonzalez Granja A Luque A CCR7 is mainly expressed in teleost gills, where it defines an IgD+IgM- B lymphocyte subset. J Immunol (2014) 192:1257–66. 10.4049/jimmunol.1302471 24353268

[30] Ramirez-Gomez F Greene W Rego K Hansen JD Costa G Kataria P Discovery and characterization of secretory IgD in rainbow trout: secretory IgD is produced through a novel splicing mechanism. J Immunol (2012) 188:1341–9. 10.4049/jimmunol.1101938 22205025

[31] Salinas I Zhang YA Sunyer JO. Mucosal immunoglobulins and B cells of teleost fish. Dev Comp Immunol (2011) 35:1346–65. 10.1016/j.dci.2011.11.009 22133710 PMC3428141

[32] Maki JL Dickerson HW. Systemic and cutaneous mucus antibody responses of channel catfish immunized against the protozoan parasite *Ichthyophthirius multifiliis*. Clin Diagn Lab Immunol (2003) 10:876–81. 10.1128/CDLI.10.5.876-881.2003 12965920 PMC193910

[33] Zhao X Findly RC Dickerson HW. Cutaneous antibody-secreting cells and B cells in a teleost fish. Dev Comp Immunol (2008) 32:500–8. 10.1016/j.dci.2007.08.009 18045689

[34] Findly RC Zhao X Noe J Camus AC Dickerson HW. B cell memory following infection and challenge of channel catfish wit*h Ichthyophthirius multifiliis*. Dev Comp Immunol (2013) 39:302–11. 10.1016/j.dci.2012.08.007 23041614

[35] Ye J Kaattari I Kaattari S. Plasmablasts and plasma cells: reconsidering teleost immune system organization. Dev Comp Immunol (2011) 35:1273–81. 10.1016/j.dci.2011.03.005 21477614

[36] Zwollo P Haines A Rosato P Gumulak-Smith J. Molecular and cellular analysis of B-cell populations in the rainbow trout using Pax5 and immunoglobulin markers. Dev Comp Immunol (2008) 32:1482–96. 10.1016/j.dci.2008.06.008 18616961 PMC2637475

[37] Zwollo P Mott K Barr M. Comparative analyses of B cell populations in trout kidney and mouse bone marrow: establishing “B cell signatures”. Dev Comp Immunol (2010) 34:1291–9. 10.1016/j.dci.2010.08.003 20705088 PMC2945407

[38] Bromage ES Kaattari IM Zwollo P Kaattari SL. Plasmablast and plasma cell production and distribution in trout immune tissues. J Immunol (2004) 173:7317–23. 10.4049/jimmunol.173.12.7317 15585855

[39] Kaattari S Bromage E Kaattari I Analysis of long-lived plasma cell production and regulation: implications for vaccine design for aquaculture. Aquaculture (2005) 246:1–9. 10.1016/j.aquaculture.2004.12.024

[40] Ye J Kaattari IM Ma C Kaattari S. The teleost humoral immune response. Fish Shellfish Immunol (2013) 35:1719–28. 10.1016/j.fsi.2013.10.015 24436975

[41] Hohn C Petrie-Hanson L. Rag1-/- mutant zebrafish demonstrate specific protection following bacterial re-exposure. PLoS One (2012) 7:e44451. 10.1371/journal.pone.0044451 22970222 PMC3435260

[42] Netea MG. Training innate immunity: the changing concept of immunological memory in innate host defence. Eur J Clin Invest (2013) 43:881–4. 10.1111/eci.12132 23869409

[43] Ye J Kaattari IM Kaattari SL. The differential dynamics of antibody subpopulation expression during affinity maturation in a teleost. Fish Shellfish Immunol (2011) 30:372–7. 10.1016/j.fsi.2010.11.013 21093593

[44] Fillatreau S Six A Magadan S Castro R Sunyer JO Boudinot P. The astonishing diversity of Ig classes and B cell repertoires in teleost fish. Front Immunol (2013) 4:28. 10.3389/fimmu.2013.00028 23408183 PMC3570791

[45] Nakashima M Kinoshita M Nakashima H Habu Y Miyazaki H Shono S Pivotal advance: characterization of mouse liver phagocytic B cells in innate immunity. J Leukoc Biol (2012) 91:537–46. 10.1189/jlb.0411214 22058423

[46] Li J Barreda DR Zhang YA Boshra H Gelman AE Lapatra S B lymphocytes from early vertebrates have potent phagocytic and microbicidal abilities. Nat Immunol (2006) 7:1116–24. 10.1038/ni1389 16980980

[47] Merad M Sathe P Helft J Miller J Mortha A. The dendritic cell lineage: ontogeny and function of dendritic cells and their subsets in the steady state and the inflamed setting. Annu Rev Immunol (2013) 31:563–604. 10.1146/annurev-immunol-020711-074950 23516985 PMC3853342

[48] Lugo-Villarino G Balla KM Stachura DL Banuelos K Werneck MB Traver D. Identification of dendritic antigen-presenting cells in the zebrafish. Proc Natl Acad Sci U S A (2010) 107:15850–5. 10.1073/pnas.1000494107 20733076 PMC2936643

[49] Bassity E Clark TG. Functional identification of dendritic cells in the teleost model, rainbow trout (*Oncorhynchus mykiss*). PLoS One (2012) 7:e33196. 10.1371/journal.pone.0033196 22427987 PMC3299753

[50] Zhu LY Lin AF Shao T Nie L Dong WR Xiang LX B cells in teleost fish act as pivotal initiating APCs in priming adaptive immunity: an evolutionary perspective on the origin of the B-1 cell subset and B7 molecules. J Immunol (2014) 192:2699–714. 10.4049/jimmunol.1301312 24532580

[51] Bakke A Glover C Krogdahk A Feeding, Digestion and Absorption of Nutrients. Amsterdam: Elservier Academic Press (2010).

[52] Nelson J Dehn M The GI Tract in Air Breathing. London: Elservier/Academic Press (2010).

[53] Mussmann R Du Pasquier L Hsu E. Is *Xenopus* IgX an analog of IgA? Eur J Immunol (1996) 26:2823–30. 10.1002/eji.1830261205 8977274

[54] Gibbons DL Spencer J. Mouse and human intestinal immunity: same ballpark, different players; different rules, same score. Mucosal Immunol (2011) 4:148–57. 10.1038/mi.2010.85 21228770

[55] Gomez D Sunyer JO Salinas I. The mucosal immune system of fish: the evolution of tolerating commensals while fighting pathogens. Fish Shellfish Immunol (2013) 35:1729–39. 10.1016/j.fsi.2013.09.032 24099804 PMC3963484

[56] Zhang T Qiu L Sun Z Wang L Zhou Z Liu R The specifically enhanced cellular immune responses in Pacific oyster (*Crassostrea gigas*) against secondary challenge with *Vibrio splendidus*. Dev Comp Immunol (2014) 45:141–50. 10.1016/j.dci.2014.02.015 24607288

[57] Ellis AE. Innate host defence mechanisms of fish against viruses and bacteria. Dev Comp Immunol (2001) 25:827–39. 10.1016/S0145-305X(01)00038-6 11602198

[58] Inami M Taverne-Thiele AJ Schroder MB Kiron V Rombout JH. Immunological differences in intestine and rectum of Atlantic cod (*Gadus morhua* L.). Fish Shellfish Immunol (2009) 26:751–9. 10.1016/j.fsi.2009.03.007 19332137

[59] Perez-Sanchez J Estensoro I Redondo MJ Calduch-Giner JA Kaushik S Sitja-Bobadilla A. Mucins as diagnostic and prognostic biomarkers in a fish-parasite model: transcriptional and functional analysis. PLoS One (2013) 8:e65457. 10.1371/journal.pone.0065457 23776483 PMC3680472

[60] Zhang YA Salinas I Sunyer JO. Recent findings on the structure and function of teleost IgT. Fish Shellfish Immunol (2011) 31:627–34. 10.1016/J.Fsi.2011.03.021 21466854 PMC3404837

[61] Ballesteros NA Castro R Abos B Saint-Jean SSR Perez-Prieto SI Tafalla C. The pyloric caeca area is a major site for IgM(+) and IgT(+) B cell recruitment in response to oral vaccination in rainbow trout. PLoS One (2013) 8:e66118. 10.1371/journal.pone.0066118 23785475 PMC3681912

[62] Rombout JH Taverne-Thiele AJ Villena MI. The gut-associated lymphoid tissue (GALT) of carp (*Cyprinus carpio* L.): an immunocytochemical analysis. Dev Comp Immunol (1993) 17:55–66. 10.1016/0145-305X(93)90015-I 8449251

[63] Davidson GA Ellis AE Secombes CJ Route of immunization influences the generation of antibody secreting cells in the gut of rainbow trout (*Oncorhynchus mykiss*). Dev Comp Immunol (1993) 17:373–6. 10.1016/0145-305X(93)90008-E 8375570

[64] Joosten PHM Tiemersma E Threels A Caumartindhieux C Rombout JHWM Oral vaccination of fish against *Vibrio anguillarum* using alginate microparticles. Fish Shellfish Immunol (1997) 7:471–85. 10.1006/Fsim.1997.0100

[65] Hebert P Ainsworth AJ Boyd B. Histological enzyme and flow cytometric analysis of channel catfish intestinal tract immune cells. Dev Comp Immunol (2002) 26:53–62. 10.1016/S0145-305X(01)00044-1 11687263

[66] Nigam AK Kumari U Mittal S Mittal AK Comparative analysis of innate immune parameters of the skin mucous secretions from certain freshwater teleosts, inhabiting different ecological niches. Fish Physiol Biochem (2012) 38:1245–56. 10.1007/s10695-012-9613-5 22350522

[67] Zilberg D Klesius PH. Quantification of immunoglobulin in the serum and mucus of channel catfish at different ages and following infection with *Edwardsiella ictaluri*. Vet Immunol Immunopathol (1997) 58:171–80. 10.1016/S0165-2427(97)00033-0 9336885

[68] Grontvedt RN Espelid S. Immunoglobulin producing cells in the spotted wolffish (*Anarhichas minor* Olafsen): localization in adults and during juvenile development. Dev Comp Immunol (2003) 27:569–78. 10.1016/S0145-305X(03)00028-4 12697313

[69] Xu DH Klesius PH Shelby RA. Cutaneous antibodies in excised skin from channel catfish, *Ictalurus punctatus* Rafinesque, immune to *Ichthyophthirius multifiliis*. J Fish Dis (2002) 25:45–52. 10.1046/j.1365-2761.2002.00339.x15813863

[70] Haugarvoll E Bjerkas I Nowak BF Hordvik I Koppang EO. Identification and characterization of a novel intraepithelial lymphoid tissue in the gills of Atlantic salmon. J Anat (2008) 213:202–9. 10.1111/j.1469-7580.2008.00943.x 19172734 PMC2526113

[71] Koppang EO Fischer U Moore L Tranulis MA Dijkstra JM Kollner B Salmonid T cells assemble in the thymus, spleen and in novel interbranchial lymphoid tissue. J Anat (2010) 217:728–39. 10.1111/j.1469-7580.2010.01305.x 20880086 PMC3039185

[72] Chen K Cerutti A. The function and regulation of immunoglobulin D. Curr Opin Immunol (2011) 23:345–52. 10.1016/j.coi.2011.01.006 21353515 PMC3109135

[73] Xu Z Gomez D Parra D Takizawa F Sunyer JO IgT plays a prominent role in gill immune response of rainbow trout. Fish Shellfish Immunol (2013) 34:1686–1686. 10.1016/j.fsi.2013.03.161

[74] von Gersdorff Jorgensen L Heinecke RD Skjodt K Rasmussen KJ Buchmann K. Experimental evidence for direct in situ binding of IgM and IgT to early trophonts of *Ichthyophthirius multifiliis* (Fouquet) in the gills of rainbow trout, *Oncorhynchus mykiss* (Walbaum). J Fish Dis (2011) 34:749–55. 10.1111/j.1365-2761.2011.01291.x 21916900

[75] Makesh M Sudheesh PS Cain KD. Systemic and mucosal immune response of rainbow trout to immunization with an attenuated *Flavobacterium psychrophilum* vaccine strain by different routes. Fish Shellfish Immunol (2015) 44:156–63. 10.1016/j.fsi.2015.02.003 25687393

[76] Tacchi L Musharrafieh R Larragoite ET Crossey K Erhardt EB Martin SA Nasal immunity is an ancient arm of the mucosal immune system of vertebrates. Nat Commun (2014) 5:5205. 10.1038/ncomms6205 25335508 PMC4321879

[77] LaPatra S Kao S Erhardt EB Salinas I. Evaluation of dual nasal delivery of infectious hematopoietic necrosis virus and enteric red mouth vaccines in rainbow trout (*Oncorhynchus mykiss*). Vaccine (2015) 33:771–6. 10.1016/j.vaccine.2014.12.055 25562788

[78] Rombout JH Yang G Kiron V. Adaptive immune responses at mucosal surfaces of teleost fish. Fish Shellfish Immunol (2014) 40:634–43. 10.1016/j.fsi.2014.08.020 25150451

[79] Min L Li-Li Z Jun-Wei G Xin-Yuan Q Yi-Jing L Di-Qiu L. Immunogenicity of *Lactobacillus*-expressing VP2 and VP3 of the infectious pancreatic necrosis virus (IPNV) in rainbow trout. Fish Shellfish Immunol (2012) 32:196–203. 10.1016/j.fsi.2011.11.015 22138084

[80] Ballesteros NA Rodriguez Saint-Jean S Perez-Prieto SI Aquilino C Tafalla C. Modulation of genes related to the recruitment of immune cells in the digestive tract of trout experimentally infected with infectious pancreatic necrosis virus (IPNV) or orally vaccinated. Dev Comp Immunol (2014) 44:195–205. 10.1016/j.dci.2013.12.009 24370535

[81] Galindo-Villegas J Mulero I Garcia-Alcazar A Munoz I Penalver-Mellado M Streitenberger S Recombinant TNF alpha as oral vaccine adjuvant protects European sea bass against vibriosis: insights into the role of the CCL25/CCR9 axis. Fish Shellfish Immunol (2013) 35:1260–71. 10.1016/J.Fsi.2013.07.046 23932985

[82] Companjen AR Florack DE Slootweg T Borst JW Rombout JH. Improved uptake of plant-derived LTB-linked proteins in carp gut and induction of specific humoral immune responses upon infeed delivery. Fish Shellfish Immunol (2006) 21:251–60. 10.1016/j.fsi.2005.12.001 16464614

[83] Shin YJ Kwon TH Seo JY Kim TJ. Oral immunization of fish against iridovirus infection using recombinant antigen produced from rice callus. Vaccine (2013) 31:5210–5. 10.1016/j.vaccine.2013.08.085 24021312

[84] dos Santos NM Taverne-Thiele JJ Barnes AC Van Muiswinkel WB Ellis AE Rombout JH. The gill is a major organ for antibody secreting cell production following direct immersion of sea bass (*Dicentrarchus labrax*, L.) in a *Photobacterium damselae ssp piscicida* bacterin: an ontogenetic study. Fish Shellfish Immunol (2001) 11:65–74. 10.1006/fsim.2000.0295 11271603

[85] Xu Z Chen CF Maod ZJ Zhud WY Detection of serum and mucosal antibody production and antibody secreting cells (ASCs) in large yellow croaker (*Pseudosciaena crocea*) following vaccination with *Vibrio harveyi* via different routes. Aquaculture (2009) 287:243–7. 10.1016/j.aquaculture.2008.10.026

[86] Esteve-Gassent MD Nielsen ME Amaro C. The kinetics of antibody production in mucus and serum of European eel (*Anguilla anguilla* L.) after vaccination against *Vibrio vulnificus*: development of a new method for antibody quantification in skin mucus. Fish Shellfish Immunol (2003) 15:51–61. 10.1016/S1050-4648(02)00138-9 12787687

[87] Lobb CJ. Secretory immunity induced in catfish, *Ictalurus punctatus*, following bath immunization. Dev Comp Immunol (1987) 11:727–38. 10.1016/0145-305X(87)90060-7 3440500

[88] Esteve-Gassent MD Fouz B Amaro C. Efficacy of a bivalent vaccine against eel diseases caused by *Vibrio vulnificus* after its administration by four different routes. Fish Shellfish Immunol (2004) 16:93–105. 10.1016/S1050-4648(03)00036-6 15123314

[89] Kai YH Wu YC Chi SC. Immune gene expressions in grouper larvae (*Epinephelus coioides*) induced by bath and oral vaccinations with inactivated betanodavirus. Fish Shellfish Immunol (2014) 40:563–9. 10.1016/j.fsi.2014.08.005 25130145

[90] Zhang Z Wu H Xiao J Wang Q Liu Q Zhang Y. Immune responses evoked by infection with *Vibrio anguillarum* in zebrafish bath-vaccinated with a live attenuated strain. Vet Immunol Immunopathol (2013) 154:138–44. 10.1016/j.vetimm.2013.05.012 23768660

[91] Iguchi K Ogawa K Nagae M Ito F The influence of rearing density on stress response and disease susceptibility of ayu (*Plecoglossus altivelis*). Aquaculture (2003) 220:515–23. 10.1016/S0044-8486(02)00626-9

[92] Sundh H Kvamme BO Fridell F Olsen RE Ellis T Taranger GL Intestinal barrier function of Atlantic salmon (*Salmo salar* L.) post smolts is reduced by common sea cage environments and suggested as a possible physiological welfare indicator. BMC Physiol (2010) 10:22. 10.1186/1472-6793-10-22 21062437 PMC2992494

[93] Niklasson L Sundh H Fridell F Taranger GL Sundell K. Disturbance of the intestinal mucosal immune system of farmed Atlantic salmon (*Salmo salar*), in response to long-term hypoxic conditions. Fish Shellfish Immunol (2011) 31:1072–80. 10.1016/j.fsi.2011.09.011 21959038

[94] Kvamme BO Gadan K Finne-Fridell F Niklasson L Sundh H Sundell K Modulation of innate immune responses in Atlantic salmon by chronic hypoxia-induced stress. Fish Shellfish Immunol (2013) 34:55–65. 10.1016/j.fsi.2012.10.006 23085636

[95] Niklasson L Sundh H Olsen RE Jutfelt F Skjodt K Nilsen TO Effects of cortisol on the intestinal mucosal immune response during cohabitant challenge with IPNV in Atlantic salmon (*Salmo salar*). PLoS One (2014) 9:e94288. 10.1371/journal.pone.0094288 24809845 PMC4014467

[96] Baumgarner BL Bharadwaj AS Inerowicz D Goodman AS Brown PB. Proteomic analysis of rainbow trout (*Oncorhynchus mykiss*) intestinal epithelia: physiological acclimation to short-term starvation. Comp Biochem Physiol Part D Genomics Proteomics (2013) 8:58–64. 10.1016/j.cbd.2012.11.001 23261852

[97] Olsen RE Sundell K Mayhew TM Myklebust R Ringø E Acute stress alters intestinal function of rainbow trout, *Oncorhynchus mykiss*. Aquaculture (2005) 250:480–95. 10.1016/j.aquaculture.2005.03.014

[98] Tacchi L Lowrey L Musharrafieh R Crossey K Larragoite ET Salinas I. Effects of transportation stress and addition of salt to transport water on the skin mucosal homeostasis of rainbow trout (). Aquaculture (2015) 435:120–7. 10.1016/j.aquaculture.2014.09.027 25705060 PMC4332845

[99] Bui P Bagherie-Lachidan M Kelly SP. Cortisol differentially alters claudin isoforms in cultured puffer fish gill epithelia. Mol Cell Endocrinol (2010) 317:120–6. 10.1016/j.mce.2009.12.002 19969041

[100] Ringø E Zhou Z He S Olsen RE Effect of stress on intestinal microbiota of Arctic charr, Atlantic salmon, rainbow trout and Atlantic cod: a review. Afr J Microbiol Res (2014) 8:609–18. 10.5897/AJMR2013.6395

[101] Suzuki Y Tasumi S Tsutsui S Okamoto M Suetake H. Molecular diversity of skin mucus lectins in fish. Comp Biochem Physiol B Biochem Mol Biol (2003) 136:723–30. 10.1016/S1096-4959(03)00178-7 14662297

[102] Easy RH Ross NW. Changes in Atlantic salmon (*Salmo salar*) epidermal mucus protein composition profiles following infection with sea lice (*Lepeophtheirus salmonis*). Comp Biochem Physiol Part D Genomics Proteomics (2009) 4:159–67. 10.1016/j.cbd.2009.02.001 20403764

[103] Caipang CM Lazado CC Brinchmann MF Rombout JH Kiron V. Differential expression of immune and stress genes in the skin of Atlantic cod (*Gadus morhua*). Comp Biochem Physiol Part D Genomics Proteomics (2011) 6:158–62. 10.1016/j.cbd.2011.01.001 21262593

[104] Vatsos IN Kotzamanis Y Henry M Angelidis P Alexis M. Monitoring stress in fish by applying image analysis to their skin mucous cells. Eur J Histochem (2010) 54:e22. 10.4081/ejh.2010.e22 20558343 PMC3167306

[105] Haond C Nolan DT Ruane NM Rotllant J Wendelaar Bonga SE. Cortisol influences the host-parasite interaction between the rainbow trout (*Oncorhynchus mykiss*) and the crustacean ectoparasite *Argulus japonicus*. Parasitology (2003) 127:551–60. 10.1017/S0031182003004116 14700191

[106] Boutin S Bernatchez L Audet C Derome N. Network analysis highlights complex interactions between pathogen, host and commensal microbiota. PLoS One (2013) 8:e84772. 10.1371/journal.pone.0084772 24376845 PMC3871659

[107] Shrimpton JM McCormick SD. Responsiveness of gill Na+/K+-ATPase to cortisol is related to gill corticosteroid receptor concentration in juvenile rainbow trout. J Exp Biol (1999) 202(Pt 8):987–95. 10085271 10.1242/jeb.202.8.987

[108] McCormick SD Endocrine control of osmoregulation in teleost fish. Integr Comp Biol (2001) 41:781–94. 10.1093/icb/41.4.781

[109] Wong CK Chan DK. Effects of cortisol on chloride cells in the gill epithelium of Japanese eel, *Anguilla japonica*. J Endocrinol (2001) 168:185–92. 10.1677/joe.0.1680185 11139782

[110] Evans DH. Cell signaling and ion transport across the fish gill epithelium. J Exp Zool (2002) 293:336–47. 10.1002/jez.10128 12115905

[111] Cui M Zhang Q Yao Z Zhang Z Zhang H Wang Y. Immunoglobulin M gene expression analysis of orange-spotted grouper, *Epinephelus coioides*, following heat shock and *Vibrio alginolyticus* challenge. Fish Shellfish Immunol (2010) 29:1060–5. 10.1016/j.fsi.2010.08.018 20816805

[112] Stolte EH Chadzinska M Przybylska D Flik G Savelkoul HF Verburg-Van Kemenade BM. The immune response differentially regulates Hsp70 and glucocorticoid receptor expression in vitro and in vivo in common carp (*Cyprinus carpio* L.). Fish Shellfish Immunol (2009) 27:9–16. 10.1016/j.fsi.2008.11.003 19061961

[113] Verburg-van Kemenade BML Nowak B Engelsma MY Weyts FAA Differential effects of cortisol on apoptosis and proliferation of carp B-lymphocytes from head kidney, spleen and blood. Fish Shellfish Immunol (1999) 9:405–15. 10.1006/fsim.1998.0197

[114] Hou Y Suzuki Y Aida K Effects of steroids on the antibody producing activity of lymphocytes in rainbow trout. Fish Sci (1999) 65:850–5.

[115] Carlson RE Anderson DP Bodammer JE In vivo cortisol administration suppresses the in vitro primary immune response of winter flounder lymphocytes. Fish Shellfish Immunol (1993) 1993:299–312. 10.1006/fsim.1993.1029

[116] Saha NR Suetake H Kikuchi K Suzuki Y Effects of steroid hormones on apoptosis and IgM production of LPS-stimulated lymphocytes in fugu *Takifugu rubripes*. Fish Sci (2006) 72:136–42. 10.1111/j.1444-2906.2006.01127.x

[117] Saha NR Usami T Suzuki Y. In vitro effects of steroid hormones on IgM-secreting cells and IgM secretion in common carp (*Cyprinus carpio*). Fish Shellfish Immunol (2004) 17:149–58. 10.1016/j.fsi.2004.01.001 15212735

[118] Saha NR Usami T Suzuki Y Seasonal changes in the immunr activities of common carp (*Cyprinus carpio*). Fish Physiol Biochem (2002) 26:379–87. 10.1023/B:FISH.0000009275.25834.67

[119] Deinzer R Schuller N. Dynamics of stress-related decrease of salivary immunoglobulin A (sIgA): relationship to symptoms of the common cold and studying behavior. Behav Med (1998) 23:161–9. 10.1080/08964289809596372 9494693

[120] Volkmann ER Weekes NY Basal SIgA and cortisol levels predict stress-related health outcomes. Stress Health (2006) 22:11–23. 10.1002/smi.1077

[121] Campos-Rodriguez R Godinez-Victoria M Abarca-Rojano E Pacheco-Yepez J Reyna-Garfias H Barbosa-Cabrera RE Stress modulates intestinal secretory immunoglobulin A. Front Integr Neurosci (2013) 7:86. 10.3389/fnint.2013.00086 24348350 PMC3845795

[122] Zachar PC Jonz MG. Neuroepithelial cells of the gill and their role in oxygen sensing. Respir Physiol Neurobiol (2012) 184:301–8. 10.1016/j.resp.2012.06.024 22772312

[123] Zaccone G Fasulo S Ainis L. Distribution patterns of the paraneuronal endocrine cells in the skin, gills and the airways of fishes as determined by immunohistochemical and histological methods. Histochem J (1994) 26:609–29. 10.1007/BF00158286 7982786

[124] Bertotto D Poltronieri C Negrato E Majolini D Radaelli G Simontacchi C Alternative matrices for cortisol measurement in fish. Aquac Res (2010) 41:1261–7. 10.1111/J.1365-2109.2009.02417.X

